# Atraumatic thoracic spinal fracture mimicking herpes zoster neuralgia: a case report

**DOI:** 10.1186/s13256-021-02897-0

**Published:** 2021-06-04

**Authors:** Liming Cao, Xiang Xiao, Shixin Du

**Affiliations:** 1 Department of Neurology, The First Affiliated Hospital of Shenzhen University, 3002 Sungang West Road, Futian District, Shenzhen, 518000 China; 2 Department of Neurology, The Third Affiliated Hospital of Shenzhen University, Shenzhen, 518000 China; 3 Department of Rehabilitation, The Third Affiliated Hospital of Shenzhen University, Shenzhen, 518000 China; 4 Department of Bone Surgery, The Third Affiliated Hospital of Shenzhen University, Shenzhen, 518000 China

**Keywords:** Ankylosing spondylitis, Spinal cord compression, Spinal fractures, Intercostal neuralgia

## Abstract

**Background:**

Intercostal neuralgia is most common in patients with herpes zoster, but it might be the initial symptom of serious diseases, such as atraumatic spinal fracture, which may lead to serious consequences if not diagnosed and treated early. Severe intercostal neuralgia is rarely reported as the first symptom of ankylosing spondylitis with atraumatic vertebral fractures.

**Case presentation:**

A 70-year-old Chinese Han man previously diagnosed with ankylosing spondylitis presented to the hospital with intense intercostal pain without trauma. The patient was initially suspected of having herpes zoster neuralgia; however, he subsequently experienced numbness and weakness of both lower limbs as well as constipation. Thoracic vertebral fracture and compression of the spinal cord were detected with magnetic resonance imaging, and he underwent emergency posterior thoracic spinal canal decompression, and intercostal neuralgia was relieved after surgery. Spinal tuberculosis and tumors were later excluded by pathological examination and follow-up results. A 6-month postoperative follow-up showed that the weakness and numbness of the left lower limb had significantly improved, and his urinary function had recovered.

**Conclusions:**

Patients with ankylosing spondylitis could develop atraumatic spinal fractures. Severe intercostal neuralgia is an early indicator of spinal fractures, and spinal magnetic resonance imaging is crucial for the diagnosis.

## Background

Intercostal neuralgia is most common in patients with herpes zoster, which may be an early manifestation of serious diseases, such as traumatic spinal fracture, which is an unexpected clinical phenomenon. The initial symptom of atraumatic spinal fracture may be intercostal neuralgia,which may have serious consequences if not diagnosed and treated early. The estimated incidence of ankylosing spondylitis (AS) in the general population is 0.1–0.5% [1] and is characterized by inflammatory back pain, radiographic sacroiliitis, excess spinal bone formation, and a high prevalence of HLA-B27 [2]. The prevalence of vertebral fractures in patients with AS is highly variable across different studies, from 0.4% [3] to 40.9% [4]. The incidence of major neurological complications following a vertebral fracture in AS is 29–91% [5]. Severe intercostal neuralgia is rarely reported as the first symptom of AS with atraumatic vertebral fractures. Hence, physicians may not immediately consider spinal fracture. This may lead to misdiagnosis or delayed diagnosis, increasing the risk of neurological complications with low recovery rates [6]. We herein report a case of atraumatic thoracic spinal fracture mimicking herpes zoster neuralgia and present a brief review of the literature to improve the accuracy of diagnosis of this condition.

## Case presentation

A 70-year-old Chinese Han man was hospitalized in August 2017 after 2 weeks of recurrent severe intercostal pain with no skin herpes or fever. He had transient incomplete intestinal obstruction and urinary retention for 2 weeks before admission. On arrival, the nature of the intercostal pain (T9–10 level) was prickling or cutting, which was initially improved with pregabalin. Thus, we suspected early herpes zoster virus-related neuralgia, which is the most common form of neuralgia in the Department of Neurology. He had smoked 20 cigarettes per day for over 20 years. He was diagnosed with AS 40 years ago. He had a history of hypertension and sequelae of cerebral hemorrhage for more than 20 years, resulting in impaired speech and right limb paralysis, and had been bedridden for many years. Moreover, he had epilepsy for approximately 15 years, benign prostatic hyperplasia for 10 years, osteoporosis for approximately 20 years, a skin ulcer on his right foot for 6 years, and chronic heart failure for 20 years. He had also undergone right hip surgery 20 years ago, but had no hereditary conditions.

### Clinical findings

Upon admission, the patient’s blood pressure was 128/60 mmHg, with coarse respiratory sounds in both lungs. He displayed clear consciousness, slurred speech, grade 3 right upper extremity muscle strength, grade 2 right lower extremity muscle strength, grade 4 left upper extremity muscle strength (which could be affected by severe body pain), grade 3 left lower extremity muscle strength, hyperalgesia in the trunk region (T9–10 level), and a negative pyramidal tract sign. His thoracolumbar spine was also rigid, resulting in limited movement.

### Diagnostic assessment

**Fig. 1:**
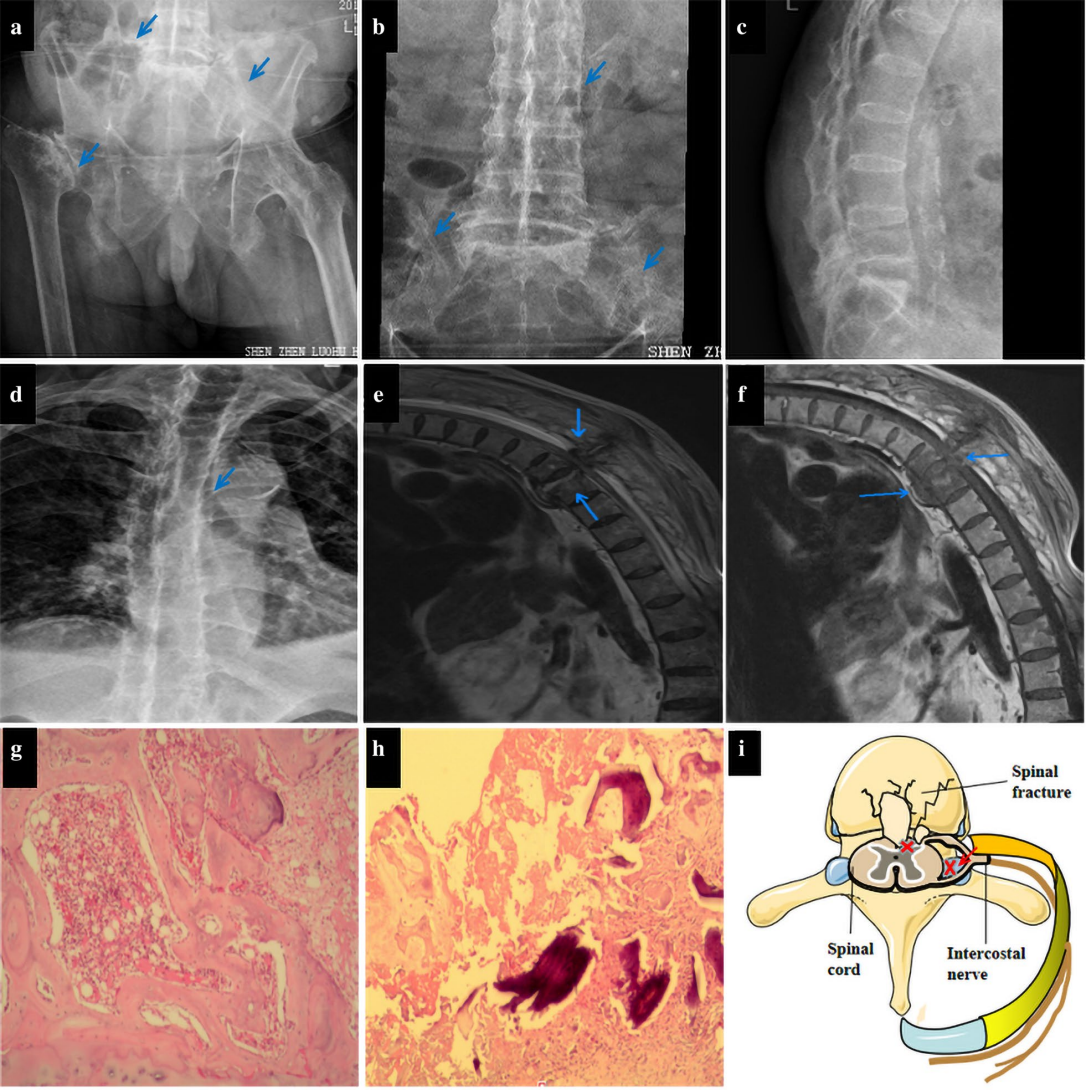
**a** Pelvic radiograph revealing bilateral sacroiliac, and left hip joints with sclerosis of articular surface, narrowing of joint space, and disappearance of partial joint space (arrows). Osteonecrosis of the right femoral head is also found. **b** Lumbar vertebra radiograph showing typical “bamboo spine” (arrow), narrowing and disappearance of the left sacroiliac joint space, and sclerosis of the articular surface. **c** Lateral view radiograph of lumbar vertebra showing reversal of the physiological curve of lumbar vertebra. **d** Thoracic spine radiograph revealing thoracic spine kyphosis, multiple synostoses in the margin of the vertebral body, and “bamboo spine” (arrow). The image is in accordance with the manifestations of ankylosing spondylitis (AS). **e**, **f** Enhanced thoracolumbar magnetic resonance imaging (MRI) revealing an extensively abnormal signal (**e**–**f**, arrows) at T9–10, destruction of the thoracic vertebra (**e–f**, arrows), and bleeding changes (**e**, arrow) within the lesions with intervertebral disc denaturation. Discontinuity of T9–10 interspinal ligament with cord-like high signal intensity in T2-weighted image; rupture of the interspinous ligament was suspected. **g**, **h** Pathological examination of the soft tissue around the abnormal thoracic vertebra revealing the presence of necrosis, dead bone fragments, proliferation of granulation tissue, fibrous connective tissue accompanied by focal hemorrhages, and inflammatory cell infiltration, with no tumor cells, no focus of spinal tuberculosis, and no fungi observed using a light microscope (hematoxylin and eosin, original magnification ×40). **i** (Graphical abstract) Schematic diagram of intercostal neuralgia caused by vertebral fracture. Fracture of the thoracic spine results in compression of the spinal cord and spinal nerve root, the latter of which extends out via the intercostal nerve

We performed supplementary examinations after his admission. His fecal occult blood test results were negative, and his levels of thyrotropin, fasting blood sugar, glycosylated hemoglobin, alpha-fetoprotein, carcinoembryonic antigen, antinuclear antibodies, and carbohydrate antigen 125 were all normal. No significant abnormalities were detected with routine blood tests, and his C-reactive protein levels were also normal. Pelvic (Fig. 1a), lumbar vertebra (Fig. 1b, c), and thoracic spine radiographs (Fig. 1d) revealed bilateral sacroiliitis and bamboo spine, which is in accordance with imaging characteristics of AS. Additionally, electrocardiography revealed myocardial ischemia.

### Therapeutic intervention

The patient was administered pregabalin (150 mg per day), and pain relief was noted on the first day; however, the treatment was not effective the next day. Gabapentin failed to control the pain, as well, and tramadol had a temporary effect. The patient experienced severe pain, which was limited by turning over. On the third day of hospitalization, he developed constipation and weakness and numbness of the left lower limb. A physical examination showed grade 2/5 muscle strength in his bilateral lower extremities, weak tendon reflexes of the lower extremities, and decreased tactile sensation below the inguinal area on both sides. We detected that the patient was presenting with symptoms of spinal cord compression, and his thoracic and lumbar vertebrae were immediately examined by enhanced magnetic resonance imaging (MRI) (Fig. 1e, f). An emergency orthopedic consultation was sought because of the severity of the spinal cord compression (which may have been caused by spinal tuberculosis or tumors). After consulting with the orthopedic specialist, the patient underwent posterior thoracic spinal canal decompression with internal fixation using a vertebral pedicle screw system under general anesthesia. The patient reported immediate relief and improvement of pain, hyperalgesia at the T9–10 level, and grade 3/5 muscle strength in the left lower limb after the operation. No acid-fast bacilli, yeast-like fungi, or bacteria were found in the smears of the thoracic vertebral lesions (the tissue sample is obtained from the thoracic spine and peripheral soft tissues) examined postoperatively. Pathological examination of the surgically excised thoracic vertebral lesions revealed inflammatory changes, but no tumor cells (Fig. 1g, h). The tests for tuberculosis bacillus deoxyribonucleic acid (by fluorescent polymerase chain reaction amplification), tuberculosis polypeptide antigen, and tuberculosis protein-specific cells (at the lesion) were also negative.

### Follow-up and outcomes

A postoperative follow-up at 1 month showed that the weakness and numbness in the lower-left limb (the muscle strength of the left lower limb was grade 3) had improved, and the patient was experiencing normal evacuation of the bladder. A postoperative follow-up at 6 months confirmed that the patient’s condition was stable. The Modified Rankin Scale score was 4. The patient was satisfied with the treatments and his recovery.

## Discussion

Intercostal neuralgia is rarely reported and easily neglected as the initial symptom of thoracic vertebral fracture following AS. Early atypical symptoms of spinal cord compression (SCC) lead to delays in diagnosis. Despite the absence of trauma, patients with AS could develop spontaneous spinal fractures,which can cause intercostal neuralgia (Fig. 1i), such as in our patient. AS patients with nontraumatic spine fractures are rare and hidden. Spinal MRI is crucial for diagnosis.

Our patient met the modification of the New York Diagnostic criteria of AS in 1984 [7]. The specific contents are as follows: (1) low back pain and stiffness for more than 3 months, which is not relieved by rest; (2) limited motion in the lumbar spine; and (3) pelvic X-ray plain films showing bilateral sacroiliitis grade III (Fig. 1a–d). Therefore, our patient could be diagnosed as having AS.

The causes of intercostal neuralgia are as follows: spinal tumor (primary and secondary) [8, 9], a lipoma on the outer edge of the rib [10], extensive idiopathic hyperosteogeny [11], breast-enlargement surgery [12], thoracic disc herniation, degenerative spinal disease, ossification of posterior longitudinal ligament, and spinal abscess. Intercostal neuralgia is rarely reported as the first symptom in AS patients with spine fractures.

Irritation of the spinal nerve root (for example, intercostal neuralgia) is an early marker of SCC, which is an early warning indicator of spinal fractures. In our case, the diagnosis was made only after the patient’s symptoms continued to worsen. In a previous study, five (20%) patients had a delayed diagnosis of over 24 hours after the injury [6]. Back pain may be masked by severe intercostal neuralgia, as in our patient. The pain could be localized, but clinicians require a detailed history, particularly in patients with cognitive or language disorders. SCC can cause autonomic nerve impairment with bladder and rectal dysfunction [13], as in our patient.

Our patient was bedridden for several years with no previous trauma. However, he suffered a spontaneous spinal fracture, which could be attributed to osteoporosis, a common feature of AS. AS-affected patients have a fivefold increase in the risk of spinal fractures [14]. Most (23/24) patients had spinal fractures and sustained low-energy injuries [6]. Low-energy trauma in patients with AS can cause spinal injury secondary to spinal fractures[15]. The incidence of spinal cord injury (SCI) in patients with AS was 11.4 times that of the general population [16]. Clinicians must pay attention to routine medical examinations and operations that could cause iatrogenic spinal fractures in fragile bones [17].

MRI is crucial for correct diagnosis as well as differential diagnosis of AS. Active inflammatory changes (sacroiliitis or bone marrow edema) could be detected by MRI [18]; a T2-weighted sequence with fat suppression for detecting active inflammatory changes (bone marrow edema) and a T1-weighted sequence for detecting postinflammatory changes, such as erosions, sclerosis, ankyloses, and fatty lesions [19]. The MRI features of AS with spinal fractures are analogous to those of spinal tuberculosis and tumors, as in our patient, and the misdiagnosis rate of AS with spinal fracture is as high as 34% [20]. AS stress fractures often occur in conjunction with thoracolumbar kyphosis or scoliosis, and destructive lesions (Andersson lesions) are found in the intervertebral disc in the fracture plane [20]. Moreover, the upper and lower endplates of the adjacent vertebral bodies tend to have extensive subchondral bone damage and irregular margins. MRI of the entire spinal column is recommended for patients with a painful spinal column after minor trauma and for those with persistent pain without trauma [21]. Computed tomography (CT) can show the fractures in detail; however, MRI is a reasonable option for exclusion of occult fractures undetected by CT. Somatosensory evoked potential and motor evoked potential are useful for identifying AS who are prone to combined neurological injury [22].

The prognosis of SCC depends on the location of the lesion, intensity and duration of SCC, and the patient’s overall health. In previous studies, 12 (50%) patients had a neurological deficit at the time of admission, and most did not recover [4]; 6.6% of AS patients with spinal fractures died during hospitalization [23]. AS patients with irritation of the spinal nerve root should undergo early intervention to prevent further SCI. Promptly identified signs and symptoms of spinal fracture following AS are important for prognosis.

As this is a case report, it has limited generalizability. Multicenter and large-sample clinical studies are also required to confirm the clinical characteristics of intercostal neuralgia and atraumatic spinal fractures in AS patients.

## Conclusion

Severe and unexplained intercostal neuralgia is an early indicator of spinal fractures, and spinal MRI is crucial for the diagnosis. Despite the absence of a history of trauma, AS patients could develop spontaneous spinal fractures. They have a high risk of fracture and need to be aware of atraumatic spinal fractures. Promptly identified signs and symptoms of spinal fracture in AS patients are important for better prognosis.

## Data Availability

Not applicable.
